# Rheological Properties of Organic Kerosene Gel Fuel

**DOI:** 10.3390/gels8080507

**Published:** 2022-08-14

**Authors:** Meng-Ge Li, Yan Wu, Qin-Liu Cao, Xin-Yi Yuan, Xiong Chen, Jun-Li Han, Wei-Tao Wu

**Affiliations:** 1 School of Mechanical Engineering, Nanjing University of Science & Technology, Nanjing 210094, China; 2 Shanghai Xinli Power Equipment Institute, China Aerospace Science and Technology Corporation, Shanghai 201109, China; 3 Beijing Institute of Electromechanical Technology, Beijing 100012, China

**Keywords:** kerosene gel, rheology, shear-thinning, thixotropy, constitutive relationship

## Abstract

Gel fuel potentially combines the advantages of solid fuel and liquid fuel due to its special rheological properties, which have essential impacts on the application of gel fuel in propulsion systems. In this paper, we study the rheological property of organic kerosene gel through a series of measurements on its viscosity as a function of the shear rate, temperature, and shear history. The measured datasets are then fitted with constitutive relationships between the viscosity and shear rate at three different levels: the power law shear-thinning model, the power law dependency on both the temperature and shear rate, and the thixotropic property. It is found that intense pre-shear could exhaust thixotropy and reduce viscosity of the kerosene gel. For the power law shear-thinning model, the consistency index increases with the gellant mass fraction, whereas the power law exponent remains constant. The dependence of viscosity on temperature could be well approximated by an empirical power law relationship. As for the thixotropic property of the kerosene gel, the fitted second-order kinetic model corresponds accurately to the viscosity at different shear rates and shear times. The constitutive models fitted in this work at different levels are consistent with each other and provide useful tools for further applications of organic kerosene gel fuel.

## 1. Introduction

Gel fuel is formed by adding gellant to liquid fuel and exists in a semi-solid physical state. It behaves as a solid when stationary and can flow and atomize similar to a liquid under shear effect, exhibiting the characteristics of both an elastoplastic solid and a non-Newtonian fluid [1,2]. These characteristics enable the gel fuel to combine the advantages of both liquid fuel and solid fuel: Safety and storability are greatly improved compared with liquid fuels, while the control of ignition and the combustion process is much more flexible compared with solid fuels due to the non-linear viscoelastic behavior of many gels under different levels of shear [3,4]. Kerosene, in particular, is the most common non-toxic liquid fuel at ordinary temperature, and its application as jet fuel in the aerospace and aviation industry has been highly matured for decades [5]. In this context, the study of the rheological characteristics of kerosene-based gel fuels has particular values for both scientific understanding and practical development of future propulsion systems [6].

The addition of gellant changes the internal structure of the base fuel at a molecular level, so the resulted gel system would have viscosity, that in most cases, is no longer immune to the shear rate. Further to the change in viscosity, being shear-thinning or shear-thickening, gels usually exhibit yield and thixotropic (typically time-dependent shear thinning) properties at a macroscopic level [7]. These properties vary with the type and content of the gellant and the formulation methods [8]. Understanding and quantification of the constitutive relationship (between the shear stress and shear strain) of the gel fuel is one of the key points to design a gel propulsion system, as it is closely coupled with organizations of flow, atomization, and combustion processes [9,10,11,12].

The non-linear relationship between viscosity and local shear rate is the most typical characteristic of non-Newtonian fluids. The most widely used constitutive model to describe this feature is likely the power law model proposed by Ostwald and de Waele, where the viscosity is determined to be related to the shear rate by both the consistency coefficient K and the flow index n. Rahimi et al. [13,14] characterized the gel fuel as a power law fluid to study the effect of rheological properties on the flow in tapered injectors and atomization characteristics. Based on the power law model, the Herschel–Bulkley (HB) model and Herschel–Bulkley extended (HBE) model were developed, taking the yield behavior and high shear condition into consideration. Ciezki et al. [15,16] and Franco et al. [17] adopted these models to treat the gel fuels for a deeper understanding of the flow behavior and spray properties. Cross [18] proposed another non-Newtonian model to describe pseudoplastic flow with asymptotic viscosities at zero ($\eta_{0}$) and infinite ($\eta_{\infty }$) shear rates, making it capable of describing a variety of fluids including dispersions, polymer melts, and polymeric solutions. Another constitutive model with special attention on the viscosities at zero and infinite shear rates is the Carreau–Yasuda (C–Y) model proposed by Bird et al. [19]. Yoon [20,21,22] employed this model to study the injection process of gel propellants. The C–Y model has more parameters than the Cross model and is the generalized form of the power law fluid model, which is well suited for shear-thinning fluids.

It has long been recognized that fluid viscosity can change with temperature, even for Newtonian fluids. Poiseuille first proposed the relationship between fluid viscosity and temperature, and the basic forms of the dependency could also be described by an exponential function, i.e., power law model. Since Arrhenius expressed the equation for temperature-dependent chemical rates in 1889, Andrade [23], Eyring [24], and other researchers proposed Arrhenius-like equations to describe the temperature-dependent viscosity. Rahimi et al. [25] measured the viscosity of gel fuels at various temperatures and formulated the influence of temperature into the parameters of the power law model, in which they found that the temperature has an insignificant effect on the gel power law exponent, whereas the consistency index reduces linearly with increasing temperature.

Beyond the instantaneous shear-rate and temperature, the viscosity of the gel is also influenced by its shear history, i.e., showing a time-dependent property (thixotropy) [26]. One commonly accepted explanation is that thixotropy originates from the build-up and breakdown of the internal structure of non-Newtonian fluid [27,28]. In the thixotropic models proposed by Moore [29], Hahn [30], and Peter [31], the time-dependent property of viscosity is formulated into a few “structural parameters”, which by themselves are functions of the shear rate and shear history. The specific expressions of the structural parameters can be obtained from the so-called thixotropic loop test, typically a series of up and down shear rate ramps, given that the gel fuel has “memory” of the shear history. Santos [32] incorporated structural parameters into the HBE model, and applications on silica gel show that the time-dependent behavior of the viscous force was well predicted.

Dullaert and Mewis [33] proposed a general structural kinetics model based on inelastic suspending media, in which contributions to the total stress are divided into an elastic (structure-dependent) part and a viscous part. The kinetic equation for the structure parameter considers the effects of both shear stress and Brownian motion on the build-up and breakdown of the internal structures. For the expressions of model function, viscous and elastic components are evaluated separately using stress jump experiments with steady and non-steady state starting conditions.

In this work, the gel fuel is formulated by adding organic gellant to kerosene-based jet fuel. Firstly, the equilibrium viscosities of kerosene gel at different temperatures and shear rates are obtained experimentally. The measured data points are used to fit corresponding model parameters, by which the constitutive relation between the viscosity, shear rate, and temperature is derived. Then, for the thixotropic property of kerosene gel, the time-dependent viscosity curves at specific shear rates are measured, through which a second-order viscoelastic model is developed. The parameters in all of the fitted models are consistent with each other, providing a reference for subsequent theoretical and numerical studies.

## 2. Results and Discussion

### 2.1. Shear-Thinning and Yield

**Figure 1:**
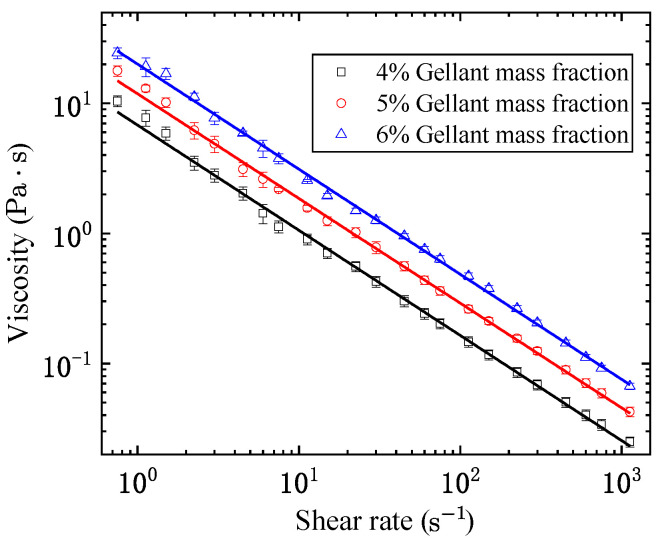
The relationship between η and $\dot{\gamma }$ of organic kerosene gel with different gellant contents after intense shear.

In this section, all of the experimental measurements are performed at a constant temperature of 298 K. Figure 1 shows the measured *η* and $\dot{\gamma }$ of organic kerosene gel with different gellant contents after intense pre-shear. Apparently, all three groups of points are distributed linearly on the log–log graph with a negative slope, which conforms to the assumption of the power law relationship given in Equation (1). In this sense, the measured data are employed to fit the parameters of Equation (1), namely, the consistency coefficient K and the power law exponent n. Table 1 lists the fitted values of K and n, along with the coefficient of determination $R^{2}$. Viscosity calculated by the fitted power law model is also plotted in Figure 1 (in lines), in comparison with the measured data (in markers). Clearly the addition of gellant enhances the viscous effect of the fluid, as the consistency coefficient K increases with the gellant mass fraction. The power law exponent n, on the other hand, hardly changes with the gellant mass fraction.

**Figure 2:**
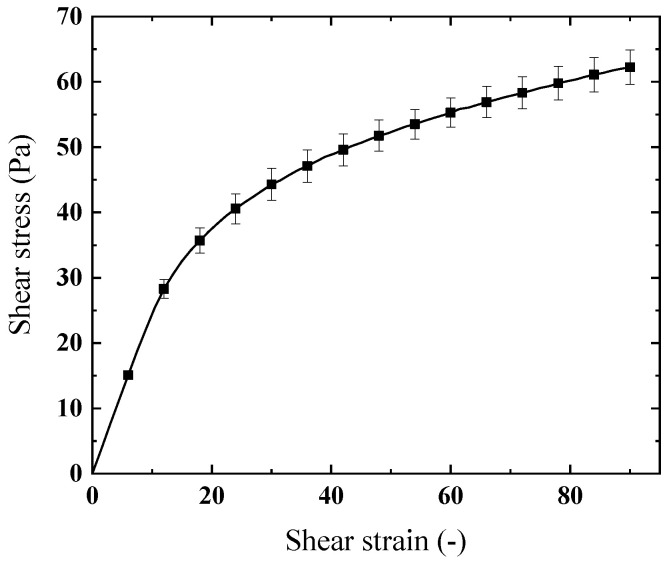
Yield stress (with standard deviation) of the kerosene gel with 5% organic gellant.

In the study of the shear-thinning properties of gel fuels, the power law model typically has good accuracy at medium shear rates, e.g., between $1{\;s}^{-1}$ and $1000{\;s}^{-1}$ as tested in this work. However, the rheological properties could deviate from the standard power law model when the shear rate falls outside the medium range due to presence of yield stress and the lower limit of viscosity. Figure 2 shows the stress–strain curve of the kerosene gel with 5% organic gellant under a low level of shear rate (typically $0.2{\;s}^{-1}$ during the test). Similar to the elastic deformation of solid materials, initially the shear strain increases linearly with the increase in shear stress. In this manner, the point where the curve stops changing linearly corresponds to the yield stress [34]. The stress–strain curve of kerosene gel is measured four times, and the average value of the yield stress ($\tau_{0}$) is 25.46 Pa with a standard deviation of 1.2 Pa.

It should be mentioned that the yield stress values are obtained by measuring the stress–strain curve with a very low level of shear rate where the gel behaves almost as a solid material. Typically, yield stress is measured to fit the thixotropic property described by Equation (2), where the equilibrium shear viscosities at various shear rates are required. For a thixotropic fluid, the equilibrium shear viscosity refers to the viscosity when the shear time is long enough until the value does not change under the action of a specific shear rate. However, in the current work, the viscosity of the kerosene gel is measured after intense pre-shear, which in fact would eliminate the yield property, and the kerosene gel can be modelled well as a “normal” power law fluid. Nevertheless, the presence of yield stress protects the solid state of the kerosene gel at rest (zero shear), which promotes the stability and storability of the gel fuel compared with liquid or slurry fuels. The improved stability permits the addition of metal particles to the gel fuel, which could substantially increase specific impulses.

### 2.2. Shear-Thinning Coupled with Temperature

**Figure 3:**
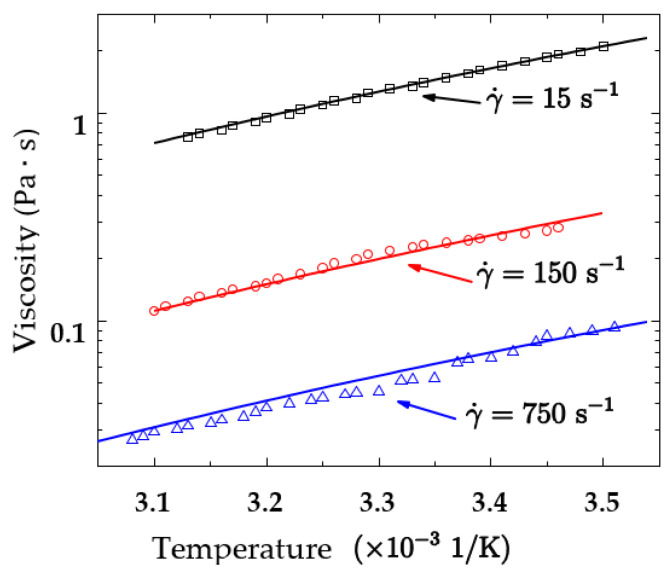
Measured (markers) and model-calculated (solid lines) viscosity of the kerosene gel with 5% organic gellant affected by both temperature and shear rate.

In this section, the temperature effect on the viscosity of the kerosene gel with 5% organic gellant is measured under three shear rates of $15{\;s}^{-1}$, $150{\;s}^{-1}$, and $750{\;s}^{-1}$. For each shear rate, the equilibrium viscosity is measured as a function of temperature, reported in the log–log plot in Figure 3. The measured data are used to fit the power law relationship between viscosity and temperature given in Equation (5); the obtained values of the parameters are as follows: $K=1.68\;\mathrm{Pa}\cdot s^{n}$, n=0.195, a=−0.0294 1/K, b=10.76. Viscosities calculated by Equation (5) are plotted as solid lines in Figure 3, which show good agreement with the experimental data (shown as markers) under all of the shear rates. Compared with the shear thinning function fitted in the previous section, where $K=11.847\;\mathrm{Pa}\cdot s^{n}$, the difference of the calculated consistency coefficients is less than 5%, as $Kexp^{aT+b}$ equals 12.399 $\mathrm{Pa}\cdot s^{n}$ with the temperature set to 298 K. It can be considered that the fitted parameters of the two constitutive models are consistent.

### 2.3. Thixotropic Loop Test

**Figure 4:**
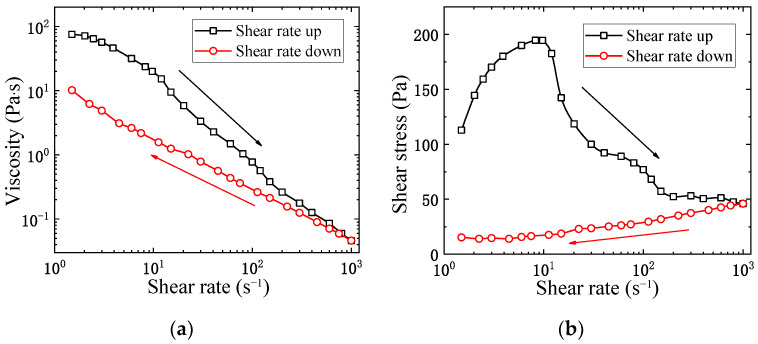
(**a**) Viscosity and (**b**) shear stress curves of the kerosene gel in the thixotropic loop test.

To show the time-dependent viscous behavior, the shear rate loop test was conducted on the kerosene gel with 5 wt% gellant, following the experimental procedure discussed in Section 2. The tracks of the measured viscosity and shear stress are shown in Figure 4.

It is not surprising that the viscosity curves during the shear rate ramp-up and ramp-down processes do not coincide with each other, clear evidence that the kerosene gel is thixotropic. In fact, the viscosities are always lower during the ramp-down process, as shown in Figure 4a. During the ramp-down process of the shear rate, the initial high shear rates already disrupt the internal structure of the gel, and then part of the shear-thinning effect is still “remembered” by the gel even after the shear rate is lowered. In the same manner, for the ramp-up process, the shear-thinning effect would be delayed by the thixotropic property. Figure 4b shows the shear stress during the loop. For Newtonian fluid with a constant viscosity, the shear stress is proportional to the shear rate, while for kerosene gel, the relationship becomes non-linear and time-dependent. Especially during the ramp-up process, the shear stress would even decrease beyond a certain value of the shear rate (around 10 $s^{-1}$ in this case), as thixotropic effects prevail in the competition with the Newtonian behavior of viscosity. During the ramp-down process, however, thixotropy is more sufficiently “exhausted” by the initial high shear rates, hence the shear stress curve remains monotonic.

**Figure 5:**
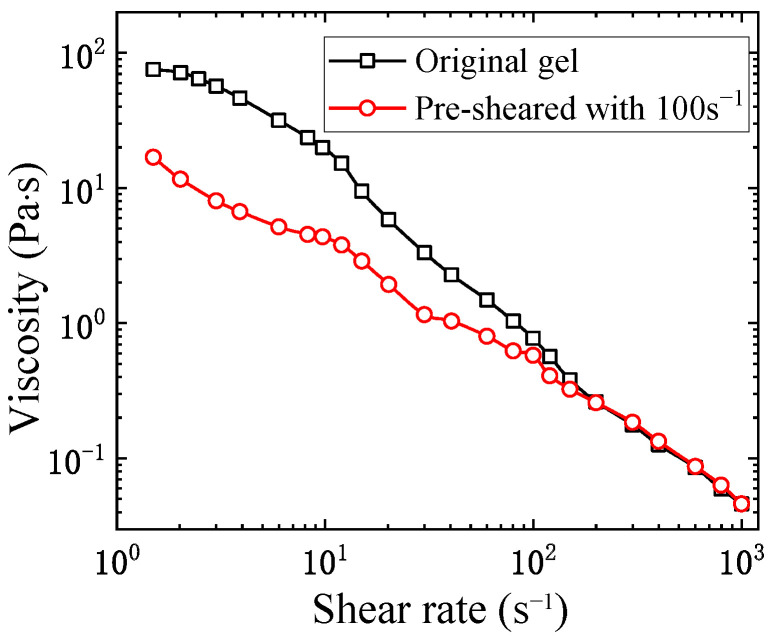
Viscosities of pre−sheared and non−pre−sheared kerosene gel under different shear rates.

As shear history has an essential influence on the viscosity of the kerosene gel fuel, which is of particular interest in engineering applications, we measured the equilibrium viscosities of two different gel samples: One pre-sheared sample with a shear rate of $100{\;s}^{-1}$ for two minutes, the other one without any pre-shearing. Figure 5 shows the records of the equilibrium viscosity values of two samples during a shear rate ramp-up process from $1.5{\;s}^{-1}$ to $1000{\;s}^{-1}$. It can be seen that in the initial stage, the gel viscosity is significantly reduced by pre-shearing. In particular, at the starting shear rate of $1.5{\;s}^{-1}$, the viscosity of the pre-sheared gel is as low as less than 25% of the “original” sample tested from zero shear. Such thixotropic behavior could be beneficial for pipeline transportation of kerosene gel, especially inside the fuel supply devices of combustors, as proper pre-shearing could significantly reduce the viscosity and hence lower the burden on the operational pressure of injectors and pumps.

### 2.4. Second-Order Dynamic Thixotropic Model

**Figure 6:**
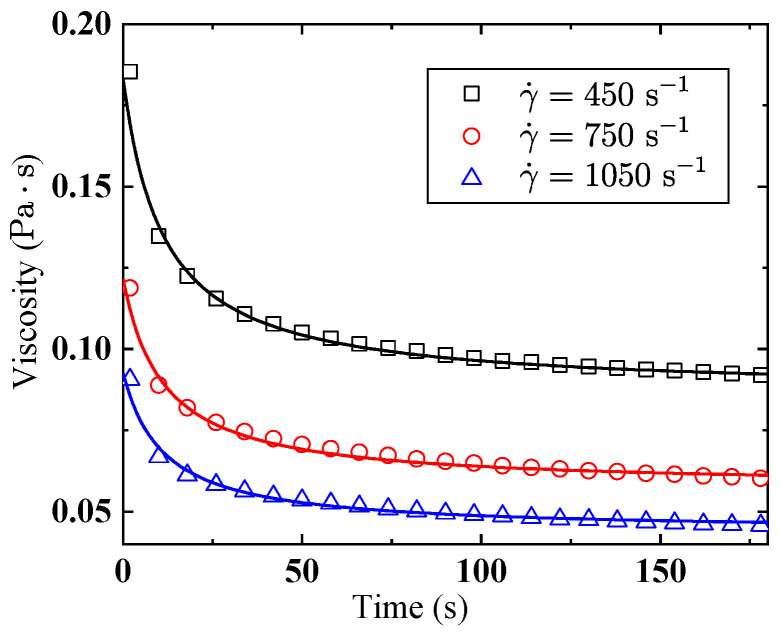
Measured (plotted in markers) and model-calculated (plotted in lines) viscosity of kerosene gel with 5% organic gellant at different shear times.

To fit a dynamic thixotropic model in the form of Equation (8), more comprehensive tests are necessary, so we measured the viscosity of kerosene gels alone with different shear times at three shear rates, namely, 450, 750, and $1050{\;s}^{-1}$, as shown by the markers in Figure 6. As shown in Table 2, the fitted consistency index (K) of kerosene gel with 5% gellant is 11.847, which translates to Equation (9) that $\lambda_{e}K=11.847$ when t is sufficiently large. It should be noted that when t=0, the expression of the structural parameter in Equation (8) is $\lambda_{e}+1/C_{1}$, which must equal unity according to the definition of the structural parameter, as the gel has not been subjected to shear effect.

As mentioned above, the structural parameter λ ranges from 1 to 0, representing the state of the gel changing from well-structured to completely sheared. The experimental results show that the time-dependent property is gradually exhausted by the continuous shear effect, while the shear-thinning property remains. In the fitting process of this work, $\lambda_{e}$ is treated as a constant value. Some studies regard $\lambda_{e}$ as a physical quantity related to the shear rate, that is, the dependence of equilibrium viscosity on the shear rates is not only due to the non-Newtonian effect but also the state of the internal structures. In this study, however, we treat *λ* as a parameter related only to the gel type. As long as the shear time is long enough, the same equilibrium state is reached at any shear rate; a higher shear rate would only shorten the time needed to reach the equilibrium state. We should also note that according to the fitted parameters (see Table 2), $\lambda_{e}$ is greater than 0. This should be attributed to the structure reconstruction property of the gel, which is balanced dynamically with the deconstruction effect of shear.

## 3. Conclusions

Better energetic performance and safer storage have always been the focus of aerospace propulsion systems. Liquid fuels with higher specific impulses, such as kerosene, are capable of continuous thrust adjustment and multiple ignitions, but are prone to leakage and lead to poor storage. The addition of suitable gellants to liquid fuels forms an intermolecular network structure that ensures the gel fuel remains in a highly viscous and stable solid-like state at rest, while the application of shearing allows the transport, atomization, and combustion similar to liquid kerosene through the destruction of the spatial structure. Thus, gel fuels are promising alternative propellants combining the advantages of both conventional liquid and solid fuels; moreover, the high viscosity of gel fuels provides a remarkably stable state for energetic metal particle suspension, thus that the specific impulse could be further increased.

In this work, we study the rheological properties of organic kerosene gel with THIXATROL ST as the gellant and anhydrous ethanol as the supplement. Viscosity of the kerosene gel is measured at different conditions of shear rate, temperature, and shear history, and the measured data are fitted to three empirical constitutive relationships, considering the shear-thinning effect, the coupled effects of shear-thinning and temperature, as well as the thixotropic property. For the thixotropic properties of the gel, a second-order kinetic model including structural parameters is employed to describe the viscosity change with the shear time. The following conclusions can be summarized:

(1) The experimental measurements suggest that the thixotropic behavior of the kerosene gel would be exhausted by continuous shear effect, degenerating into a standard shear-thinning fluid with relatively simple mechanical behavior. This is beneficial to not only the study of its constitutive relationship, but also the engineering application where the friction of transportation is one of the major concerns.
(2) After sufficient pre-shearing, the shear-thinning property could be approximated by a power law relationship between the equilibrium viscosity and the shear rate, where the consistency index K increases with the gellant mass fraction, while the power law exponent n remains almost constant.
(3) A constitutive model of the viscosity affected by both temperature and shear rate is fitted. The fitted model can accurately predict the viscosity of kerosene gel affected at a variety of temperatures and shear rates. The difference in the fitted consistency indexes between this model and the previous shear-thinning model is less than 5%.
(4) The time-dependent behavior of shear-thinning properties is investigated by thixotropic loops, based on which a second-order kinetic model is fitted. The expression of the model is consistent with the two power law relationships fitted previously, and the introduction of a structural parameter enables a reasonable prediction of the thixotropic characteristics of the kerosene gel.

## 4. Gel Preparation and Measurement Method

### 4.1. Materials and Instruments

The gellant used for the formulation of the organic kerosene gel is THIXATROL ST. Anhydrous ethanol is also added as an adjuvant to improve the quality and stability of organic kerosene-based gels and significantly increase the viscosity of the gels. An AD200L-P laboratory dispersion homogenizer is used to fully mix the basic fluid, gellant, and adjuvant. The speed of the homogenizer ranges from 300 to 21,000 RPM (revolutions per minute). After the formulation of the gel, viscosities at given conditions are measured by a DV3THB cone-plate rheometer. The rheometer adopts two different cones (namely, CPE-40 with a cone diameter of 4.8 cm and an angle of 8° and CPE-52 with a cone diameter of 2.4 cm and an angle of 3°) to provide a shear rate range of $0.02\sim 2000{\;s}^{-1}$. During the measurement, the temperature of the kerosene gel is maintained at certain constant values by a DC0506 water bath. The inlet and outlet of the water bath are connected to the sample vessel of the rheometer.

**Figure 7:**
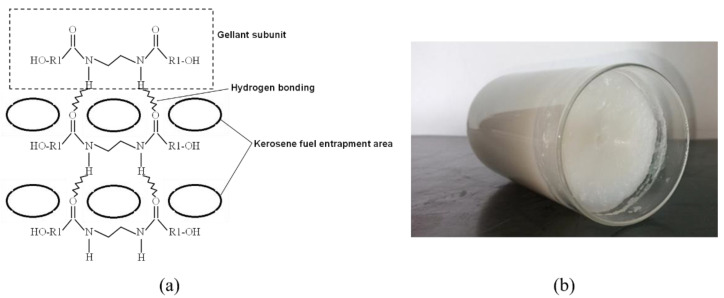
(**a**) Schematic of the THIXATROL ST gellant and kerosene fuel network structure (Reproduced with permission from Ref. [35], published by Fuel Processing Technology, 2013); (**b**) sample of organic kerosene gel fuel in a static state.

The specific steps of preparing organic kerosene gel are as follows: first, heat the kerosene to 50 °C, then add a certain amount of THIXATROL ST and anhydrous ethanol to the kerosene according to the mass fractions of the components. As shown in Table 3, three different kerosene gels are prepared and measured in this work. The gelation of organic kerosene gel is achieved through a network structure formed by self-assembly via hydrogen bonding between the well-dispersed adjacent gellant subunit in kerosene fuel, as depicted in Figure 7a. After five-minutes of a water bath and high-speed dispersal of the components, well-mixed kerosene gel is obtained. Figure 7b shows the sample with 5 wt% of THIXATROL ST; at room temperature, it is in solid state at rest.

### 4.2. Formulation of Constitutive Models

The addition of the gellant makes the viscosity of the resultant gel vary with the shear rate, i.e., exhibiting non-Newtonian behavior. The most widely used constitutive equation for non-Newtonian fluids is the power law model given by Ostwald and de Waele [36]:

(1) $$\eta =K\cdot {\dot{\gamma }}^{n-1},$$

where *η* is the shear viscosity of the gel, $\dot{\gamma }$ is the shear rate, *K* is the consistency index, and *n* is the power law exponent indicating the intensity of the shear rate dependency. As a special case that *n* = 1, it regresses to Newtonian fluid, as viscosity is independent of the shear rate. For more general non-Newtonian fluids, when *n* < 1, the viscosity decreases with the increase in the shear rate, showing the shear-thinning characteristic, while in cases where n>1, it shows a shear-thickening characteristic.

According to the original form of Equation (1), when the shear rate tends to 0, the shear-thinning (negative index on $\dot{\gamma }$) viscosity would tend to infinity; at the same time, viscosity would tend to 0 at a very high level of the shear rate (approaching infinity). As neither of these situations corresponds to physical reality, the Herschel–Bulkley Extended (HBE) model is developed to correct the power law model by considering the yield stress and the lower limit of viscosity [37]:

(2) $$\eta =\tau_{0}/\dot{\gamma }+K\cdot {\dot{\gamma }}^{n-1}+\eta_{\infty },$$

where $\tau_{0}$ is the yield stress, and $\eta_{\infty }$ is the viscosity when the shear rate tends to infinity.

When the temperature effect is considered, according to [38], fluid viscosity can be given as follows:

(3) $$\eta \left(T,\dot{\gamma }\right)=\eta_{T}\left(T\right)\cdot \eta_{\dot{\gamma }}\left(\dot{\gamma }\right),$$

according to the Andrade–Eyring law (viscosity and temperature have an exponential relationship), $\eta_{T}\left(T\right)$ can be written as follows:

(4) $$\eta_{T}\left(T\right)=K_{1}exp^{aT+b},$$

since $\eta_{\dot{\gamma }}\left(\dot{\gamma }\right)$ can also be described by a power law model, see Equation (1), $\eta \left(T,\dot{\gamma }\right)$ has the following expression:

(5) $$\eta \left(T,\dot{\gamma }\right)=K{\dot{\gamma }}^{n-1}exp^{aT+b},$$

where *K*, *a*, and *b* are model coefficients whose values can be fitted from experimental data. Compared with Equation (1), Equation (5) corresponds to a more general constitutive relationship, incorporating the temperature effect into the power law model of non-Newtonian fluid viscosity.

Beyond the non-Newtonian viscous behavior, the thixotropic nature of the gel often leads to a time-dependent shear-thinning property. For models based on structural dynamics, the thixotropy is derived from the establishment and destruction of the internal structures, whose impact on viscosity, or more generally the shear stress, is characterized by a so-called internal structure parameter λ. If the gel has not been subjected to shear effect, λ=1, whereas after the gel is sheared for a sufficiently long time (at a certain shear rate) that the viscosity no longer changes, the structure parameter at this point is the equilibrium structure parameter, $\lambda =\lambda_{e}$. In this sense, the physical value of λ should be bounded between 0 and 1 [32,39].

Assume that the decay of the structure parameter over time follows the second-order viscoelastic equation:

(6) $$\frac{d\lambda }{dt}=k_{1}{\left(\lambda -\lambda_{e}\right)}^{2},$$

where *k*_1_ is the rate constant, and the structure parameter $\lambda \geq \lambda_{e}$. By integrating Equation (6) over time, we have

(7) $$\lambda =\lambda_{e}+\frac{1}{k_{1}t+C_{1}},$$

where $C_{1}$ is the integral constant value. Specifically, for the fluids whose shear-thinning property is modelled by a power law, multiply Equation (5) by the structure parameter, and the time-dependent constitutive equation is given by

(8) $$\eta =\left(\lambda_{e}+\frac{1}{k_{1}t+C_{1}}\right)K{\dot{\gamma }}^{n-1}.$$

According to Equation (8), after shearing for a sufficiently long time (at a constant shear rate), the viscosity of the thixotropic fluid approaches its steady-state:

(9) $$\eta =\lambda_{e}K{\dot{\gamma }}^{n-1}.$$

Equation (9) gives the “equilibrium” viscosity of thixotropic fluid, whose physical significance is that (strong) shear for a sufficiently long time would eliminate the explicit time-dependent terms in the constitutive equation, turning the thixotropic fluid into normal shear-thinning fluid as described by Equation (1).

In the viscosity measurements in this work, each shear rate was applied for a sufficiently long time to ensure that the apparent viscosity of the gel no longer changed with time. For the measurement of the shear-thinning property of the kerosene gel, adopt the high shear rate of $1000\;s^{-1}$ on the kerosene gel first, and then lower the shear rate to the pre-defined value and record the reading of the rheometer as the equilibrium viscosity of the kerosene gel at the given shear rate. To control random errors, the reported values of viscosity are averaged by three measurements for each group of experimental parameters.

**Figure 8:**
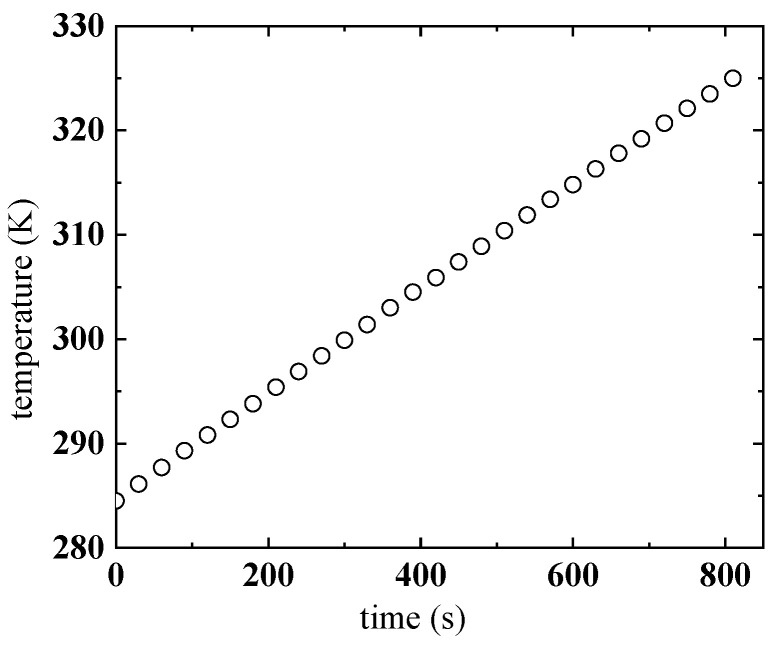
Record of the temperature rise of the water bath.

Kerosene gel with 5 wt% gellant is selected for the measurement of the viscosity affected by both the temperature and shear rate, the mass fraction of other components can be found in Table 3. During the measurement, first keep the temperature of the water bath at a low level (about 285 K), and pre-shear the kerosene gel until the viscosity no longer changes with time to eliminate the thixotropy. Then, adjust the shear rate to the desired constant value, and let the water bath gradually heat up the system to the given temperature. Figure 8 shows the temperature rise curve of the water bath; it can be seen that temperature rises at an almost constant rate of 0.05 K/s. Viscosity is recorded after both the shear rate and temperature are stabilized.

The viscosity of thixotropic fluids changes with the shear time even under a constant shear rate. The shear rate loop test is commonly used to determine whether the fluid is thixotropic: If there is an obvious difference between the viscosity curves obtained from the low-to-high path and high-to-low path of the shear rate, we assume that the fluid exhibits thixotropy. For the shear rate loop test of the kerosene gel, on the low-to-high path (increasing shear rate), the shear rate is increased from $1.5{\;s}^{-1}$ to $1000{\;s}^{-1}$, where it is then restored back to $1.5{\;s}^{-1}$ to finish the high-to-low path (shear rate decreases). During each path of the loop, the equilibrium viscosity and shear stress are recorded.

According to Equation (9), the thixotropy of the gel fuel is exhausted when the shear time is sufficiently long, turning the gel fuel into a normal power law fluid. Taking advantage of such behavior, the first step of the measurement procedure is to pre-shear the gel fuel at a high shear rate and to then record the viscosity value of this thixotropy-eliminated gel fuel at various shear rates. It should be noted that the fitted parameter here is the product of the equilibrium structure parameter and the consistency index ($\lambda_{e}K$); other parameters in Equation (8) are obtained through the shear rate loop test.

## Figures and Tables

**Table 1:** Parameters in the power law model for organic kerosene gel after intense shear.

Gellant Mass Fraction	$K\;(\mathrm{Pa}\cdot s^{n})$	n	*R* ^2^
4%	6.804	0.191	0.991
5%	11.847	0.195	0.991
6%	20.042	0.192	0.990

**Table 2:** Fitted parameters of the second-order thixotropic model based on Equation (8).

$\lambda_{e}$	$k_{1}$	$C_{1}$	K	n
0.4725	0.1696	1.8957	25.073	0.195

**Table 3:** The components of organic kerosene gel.

Kerosene (wt%)	THIXATROL ST (wt%)	Anhydrous Ethanol (wt%)
92	4	4
90	5	5
88	6	6
